# OH^−^ and H_3_O^+^ Diffusion in Model AEMs and PEMs at Low Hydration: Insights from *Ab Initio* Molecular Dynamics

**DOI:** 10.3390/membranes11050355

**Published:** 2021-05-12

**Authors:** Tamar Zelovich, Mark E. Tuckerman

**Affiliations:** 1 Department of Chemistry, New York University (NYU), New York 10003, NY, USA; 2 Courant Institute of Mathematical Sciences, New York University (NYU), New York, NY 10012, USA; 3 NYU-ECNU Center for Computational Chemistry at NYU Shanghai, 3663 Zhongshan Rd. North, Shanghai 200062, China

**Keywords:** anion-exchange membrane, proton exchange membranes, hydroxide diffusion mechanisms, hydronium diffusion mechanisms, low hydration, *ab initio* molecular dynamics, nano-confined structures

## Abstract

Fuel cell-based anion-exchange membranes (AEMs) and proton exchange membranes (PEMs) are considered to have great potential as cost-effective, clean energy conversion devices. However, a fundamental atomistic understanding of the hydroxide and hydronium diffusion mechanisms in the AEM and PEM environment is an ongoing challenge. In this work, we aim to identify the fundamental atomistic steps governing hydroxide and hydronium transport phenomena. The motivation of this work lies in the fact that elucidating the key design differences between the hydroxide and hydronium diffusion mechanisms will play an important role in the discovery and determination of key design principles for the synthesis of new membrane materials with high ion conductivity for use in emerging fuel cell technologies. To this end, *ab initio* molecular dynamics simulations are presented to explore hydroxide and hydronium ion solvation complexes and diffusion mechanisms in the model AEM and PEM systems at low hydration in confined environments. We find that hydroxide diffusion in AEMs is mostly vehicular, while hydronium diffusion in model PEMs is structural. Furthermore, we find that the region between each pair of cations in AEMs creates a bottleneck for hydroxide diffusion, leading to a suppression of diffusivity, while the anions in PEMs become active participants in the hydronium diffusion, suggesting that the presence of the anions in model PEMs could potentially promote hydronium diffusion.

## 1. Introduction

Fuel cell-based anion exchange membranes (AEMs) constitute some of the cleanest low-cost electrochemical devices [1,2,3,4,5]. This is largely due to the use of an alkaline environment, which eliminates the need for precious metal catalysts [2,3,5,6,7,8,9,10,11]. However, enhancing hydroxide ion conductivity and membrane stability remains a key hurdle to realizing the potential of AEM fuel cells [2,3,5]. Compared to AEMs, fuel cell-based proton exchange membranes (PEMs) have received far more attention over the last decade due to their promise in technologies for clean and efficient power generation [12,13,14,15,16,17,18]. The morphology, structure, and diffusion mechanism of hydronium ions in these devices have been investigated under a variety of environmental conditions. The sulfonate anionic functional end group (${\mathrm{SO}}_{3}^{-}$) is one of the most widely used groups in PEM fuel cell devices [14,19,20,21,22,23,24,25,26,27,28]. Despite the abundance of studies in the field of PEMs, ongoing discussion continues about the role of the protonation state of ${\mathrm{SO}}_{3}^{-}$ in the underlying atomistic mechanism governing the hydronium ion diffusion process. 

In recent years, nano-confined structures have been exploited in the study of cost-effective and reliable polymer architectures for electrochemical devices [29,30,31,32,33,34,35,36,37]. Understanding the water structure and the behavior of ions in these confined structures is essential to gain insight into the ion diffusion mechanisms. Hydronium and hydroxide ions diffusion mechanisms are well-studied phenomena in bulk aqueous solution and have been shown to be substantially different from one another at a fundamental level [38,39,40,41,42,43,44,45,46,47,48]. Furthermore, recent studies suggest that, based on entropic and structural considerations, the hydronium concentration at surfaces is higher than that of hydroxide ions [49,50]. These results strengthen the hypothesis that the diffusion mechanisms of hydronium and hydroxide ions in nano-confined structures are significantly different and, therefore, should be further explored in depth. 

Recently, we employed fully atomistic *ab initio* molecular dynamics (AIMD) [51] simulations to study hydroxide and hydronium ion diffusion in model AEMs and PEMs using nano-confined environments. We found that the water molecules in these models exhibit intriguing and unusual structures that depend on the shape and size of the confining volume, the hydration level, and the cation spacing. Specifically, for systems in which two-dimensional graphane bilayers (GBs) are used to mimics the actual polymer architectures [52,53,54,55], under low hydration values (λ < 5, where λ is the number of water molecules per cation/anion), the water assumes a non-uniform distribution, which is characterized by the formation of void areas throughout the system [53,55]. Consequently, the local hydroxide and hydronium diffusion mechanisms were shown to differ from their respective bulk solution mechanisms [38,39,40,41,42,43,44,45,46,47,48] in ways that are strongly dependent on the water structure. We find these unusual water distributions to be the key factor affecting the hydroxide and hydronium ions diffusion mechanisms. 

In this study, we aim to elucidate the differences in the atomistic details of the hydroxide and hydronium ion diffusion mechanisms in AEMs and PEMs in confined environments and under low hydration conditions. To this end, we apply a similar protocol from our previous studies [52,53,54,55] to explore hydroxide and hydronium diffusion in architecturally distinct AEMs and PEMs, employing nano-confined structures. We employ fully atomistic AIMD [51] to simulate the molecular behavior, solvation patterns, and ion diffusion mechanisms within the model AEMs and PEMs. We find that for AEMs, hydroxide ion diffusion is mostly vehicular, while for PEMs, hydronium diffusion is structural rather than vehicular. Furthermore, the region between each pair of cations in AEMs was found to create a bottleneck for hydroxide diffusion, leading to a suppression of hydroxide ion diffusivity, while the anions in PEMs become active participants in the hydronium ion diffusion, suggesting that under the right hydration conditions, the presence of the anions in model PEMs is likely to promote, rather than suppress, hydronium diffusion. Importantly, identifying the atomistic differences in the hydroxide and hydronium diffusion mechanisms present in the nano-confined environment of model AEMs and PEMs will help reveal key principles for creating new stable membrane materials with high ion conductivity for use in emerging fuel cell technologies.

## 2. Description of Systems

**Figure 1:**
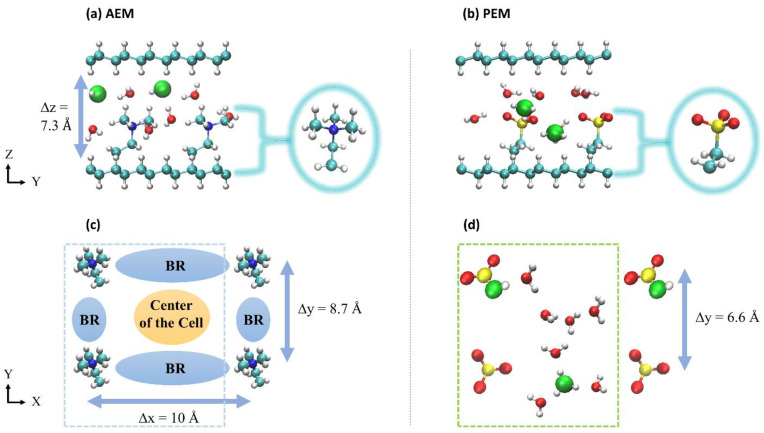
Upper figures: a side perspective of the atomistic graphane bilayer (GB) models, each consisting of two graphane sheets and a number of water molecules determined by the value of λ. (**a**) The model anion exchange membranes (AEMs) consist of two tetramethylammonium (TMA) cations and two OH^−^ ions. (**b**) The model proton exchange membranes (PEMs) consist of two ${\mathrm{SO}}_{3}^{-}$ anions and two H_3_O^+^ ions. Lower figures: The view of a typical (**c**) AEM and (**d**) PEM cells along the *z*-direction (with the upper and lower graphane sheets removed for clarity), demonstrating the cation and anion spacing in the *x*- and *y*-axes. For the AEM model, the blue and orange areas indicate the bottleneck regions (BRs) and the center of the cell regions, respectively. The water molecules were removed for better views of the BR. The green and blue rectangles show the primitive simulation cell of the system. The turquoise arrows demonstrate the polymer electrolyte cation spacing in the *x*, *y*, and *z* directions. The red, white, turquoise, blue, and yellow spheres represent O, H, C, N, and S atoms, respectively.

A typical AEM or PEM model requires a polymer backbone [1], a cationic/anionic group to ensure charge neutrality [56], and a molecular tether that connects the two via covalent bonds. Hydroxide and hydronium ions serve as charge carriers, respectively in AEMs and PEMs, and these systems are then solvated with water molecules [57]. In previous studies, we explored different GB systems as mimics of different AEM and PEM environments [52,53,54,55] in which the nanoconfined structure in these systems model the layered arrangement recently reported in References [16,57]. Of the various systems studied, we ultimately chose two representative examples that best captured the hydroxide and hydronium ion diffusion mechanisms described above. Each system contains two identical graphane layers aligned in the *xy*-plane and a number of water molecules (the hydration level) chosen to model the experimentally relevant hydration values from References [16,57]. For the AEM model, the system contains two tetramethylammonium (TMA) cations and two hydroxide ions, while for the PEM model, the system contains two ${\mathrm{SO}}_{3}^{-}$ anions and two hydronium ions. The hydroxide and hydronium ion oxygen cores are denoted O*_1_ and O*_2_ throughout the paper. The two cations/anions are attached by (CH_2_)_2_ linkers to fixed points in the GBs but are otherwise free to move in the aqueous solution. The two attachment points define the polymer electrolyte cation/anion spacing in the *x* and *y* directions (see Figure 1). As a result, the simulation cell is partitioned into an open region in the center of the cell and constricted regions between the cations/anions, which we refer to as bottleneck regions (BRs) in the discussions of the AEM models. Based on References [16,57], the tunable parameters for the two systems are: (i) the hydration number, *λ*, chosen to be 4 or 3 for both the AEM and PEM models, respectively, (ii) the polymer electrolyte cation/anion spacing in the *x* direction, Δ*x*, as measured between two nitrogen (AEM) or sulfur (PEM) atoms, which is fixed at 10 Å for the two systems, (iii) the polymer electrolyte cation/anion spacing in the *y* direction, Δ*y*, as measured between two nitrogen (AEM) or sulfur (PEM) atoms, which is fixed at 8.7 Å and 6.6 Å for the model AEM and PEM systems, respectively, and (iv) the distance between the two carbon sheets, Δ*z*, fixed at 7.3 Å for the two systems. The distance Δ*z* is calculated as the distance between the hydrogen atoms on the inner surfaces of the graphane layers and is determined by λ, with a lower bound determined by the height of the cations/anions. These AEM and PEM models were previously referred to as systems a4 and λ3 in References [53,55], respectively.

## 3. Computational Method

AIMD simulations [51] were performed on the aforementioned systems using the CPMD code [58,59]. The two systems were first equilibrated at room temperature using a massive Nosé–Hoover chain thermostat [60], followed by 15–20 ps of canonical (NVT) dynamics, also using a massive Nosé–Hoover chain thermostat, and finally ~80 ps of microcanonical (NVE) dynamics. Dispersion forces were accounted for using the Dispersion-Corrected Atomic Core Pseudopotential (DCACP) scheme [61,62] within the Kohn–Sham formulation of Density Functional Theory using the B-LYP exchange-correlation functional [63,64]. The B-LYP+DCACP has been selected for this study, as it has previously been shown to produce satisfactory results for water-acene interactions [65], liquid water [66], and hydronium diffusion in bulk water [38,39,40,67] A detailed description of the construction of the initial structures and of the computational methodology can be found in the Supplementary Information (SI) and in References [52,53,54,55]. 

## 4. Result

### 4.1. Water Distribution

**Figure 2:**
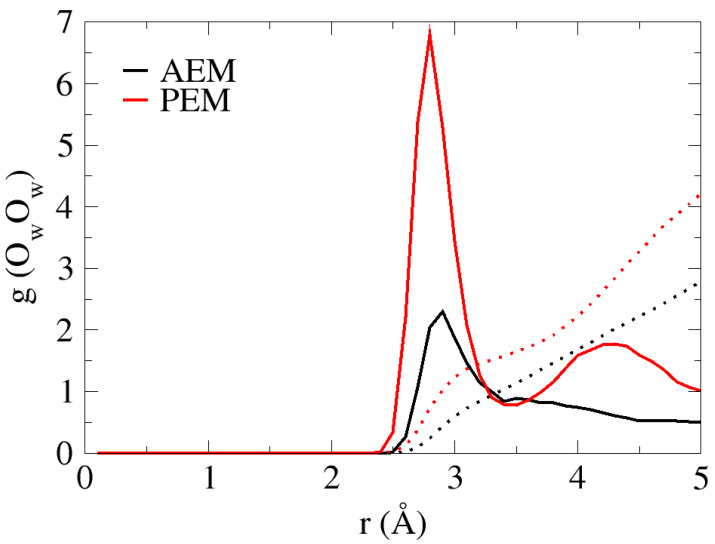
O_w_O_w_ Radial distribution functions (RDFs) of the two systems (black and red curves for AEM and PEM models, respectively). Colored dotted lines show the coordination numbers (CNs) for each system.

Inspection of the AIMD trajectories reveals that under low hydration conditions, the water distribution is non-uniform in both the AEM and PEM systems. The non-uniformity is pronounced in void areas throughout the system, in which all water molecules in the system are in contact with some part of the “membrane” and inhomogeneous throughout the system. Unlike in bulk solution, in which the water oxygen has an average of a fourfold-tetrahedral coordination pattern [38,39], the non-uniform water distribution results in a first solvation shell that oscillates between zero, one and two for the water oxygens. In order to demonstrate the existence of water-oxygen solvation shells, we present the O_w_O_w_ radial distribution functions (RDFs) and coordination numbers (CNs) for the two systems in Figure 2. As shown for both cases, the first peak located at ~2.8 Å corresponds roughly to that of bulk water. However, as a result of the non-uniform water distribution, the CN values for the first and second solvation shells are lower than those in the bulk [38,39], with values of ~1 and ~2.7 for the AEM model, and ~1.5 and ~4 for the PEM model, respectively. 

### 4.2. Hydroxide Ion Diffusion in the AEM Model System 

#### 4.2.1. ${\mathrm{OH}}^{-}$ Solvation Structure and Dynamical Properties

**Figure 3:**
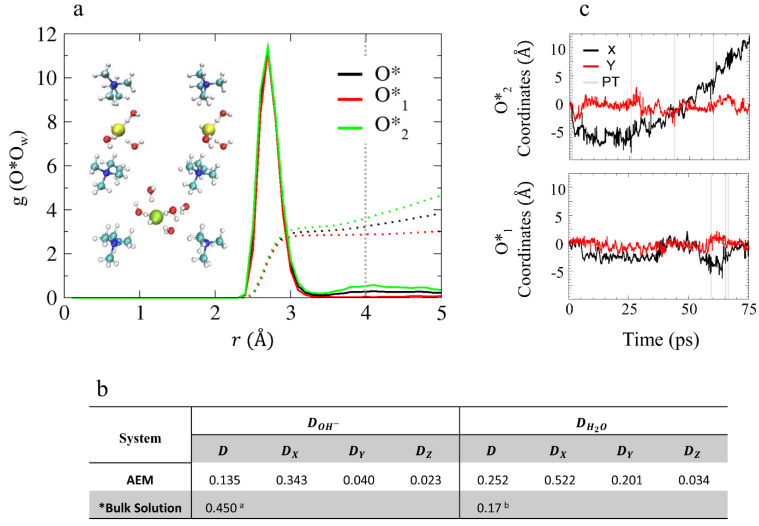
(**a**) O*O_w_ RDFs of O*, O*_1_ and O*_2_ (black, red, and green, respectively), for model AEM. The colored dotted lines represent the obtained CNs and the dotted gray line represents the hydroxide oxygen second solvation shell expected location. Inset: snapshots of hydroxide ion solvation structures obtained from AIMD trajectories that demonstrate the distinct hydroxide ion solvation shells. Red, white, turquoise, and blue spheres represent O, H, C, and N atoms, respectively. Yellow and green spheres show the position of O*_1_ and O*_2_, respectively. (**b**) Diffusion constants of the AEM model obtained from the slope of the Mean Square Displacement (MSD) in units of Å^2^/ps (MSD plots are presented in SI). * Results taken from: ^a^ Reference [45]; and ^b^ Reference [68] using the B-LYP functional. (**c**) Hydroxide ion oxygen coordinates as a function of time (black and red curves for *x* and *y* coordinates, respectively) for O*_1_ and for O*_2_ during the simulations for model AEM, with proton transfer (PT) events labeled using gray lines (excluding rattling). Figures (**a**,**c**) taken with permission from Reference [53]. Copyright 2019 American Chemical Society.

As mentioned in the previous section, the water distribution in these low hydration models is non-uniform. Specifically, for the AEM model, the non-uniformity refers to the formation of separated water clusters in the vicinity of each OH^−^ by roughly 4 Å. As a result, the two hydroxide oxygens in the AEM model may not share the same environment or have the same CN. Therefore, we find it useful to refer to the two hydroxide ions as two different species, O*_1_ and O*_2_, and to explore the O*O_w_ RDFs and CNs for the pair (O*), O*_1_, and O*_2_ separately (see Figure 3a). The first solvation shell of the hydroxide oxygen is located at 2.7 Å, as was previously shown for bulk solution [38,39,40,41,42,43,44,45,46] and contains three water oxygens (as opposed to four water oxygens as was observed in bulk solution). The CN of the second solvation shell (located at ~4 Å in bulk solution [38,39,40,41,42,43,44,45,46]) shows that O*_1_ is missing a second solvation shell while O*_2_ has a second solvation shell of two water oxygens (see inset of Figure 3a for example). It is well known that the hydroxide ion diffusion mechanism is strongly affected by the solvation structure of OH^–^ [38,39,40,41,42,43,44,45,46]. While in bulk solution, the hydroxide ions typically share a similar solvation patterns, which results in a similar diffusion mechanism in this low-hydration AEM model, the heterogeneity of the environments of each hydroxide suggests that over the time scales of the simulations, each OH^–^ might be governed by different diffusion mechanisms, which we discuss next.

The GB structures have a unique confined geometry that influences the mobility of the waters and hydroxide ions differently along each axis [52]. Therefore, in order to gain a better understanding of the water and hydroxide ion diffusion processes in these structures, we calculate diffusion coefficients along each of the axes separately (see Figure 3b), plot the coordinates of the OH^–^ oxygens as a function of time along the *x*- and *y*-axes (the coordinates in the *z*-axis are not presented as their contribution to the total diffusion is negligibly), and label proton transfer (PT) events with gray lines (see Figure 3c). As PT events represent a change in the hydroxide oxygen identity, they are used to identify different hydroxide ion diffusion mechanisms. While a sharp jump in the OH^–^ oxygens coordinates, caused by a PT event, is associated with structural diffusion [41,42,43,44,45,46] usually known as “Grotthuss diffusion”, a continuous change in the OH^–^ oxygen coordinates is associate with a vehicular diffusion [52]. As shown in Figure 3b, the hydroxide ion diffuses along the *x*-axis with a diffusion coefficient of 0.343 Å^2^/ps. However, according to Figure 3c, only O*_2_ is diffusive while O*_1_ is non-diffusive. The lack of PT events along the trajectory suggests that the diffusion of O*_2_ is mainly vehicular. 

Based on the results presented in Section 4.2.1, we find that the OH^–^ ions diffuse only when they have a second solvation shell of at least one water oxygen. The second solvation shell provides the necessary hydration that requires for the OH^–^ to shift the competition between its hydrophobic repulsion from the cation and its electrostatic attraction to the cation, which results in vehicular diffusion [38,39,40,41,42,43,44,45,46,47,48]. Hence, we find that the presence of a second solvation shell promotes vehicular diffusion in the case of O*_2,_ while the absence of a second solvation shell suppresses OH^–^ diffusion as seen for O*_1_. 

#### 4.2.2. OH^–^ Diffusion Mechanism 

**Figure 4:**
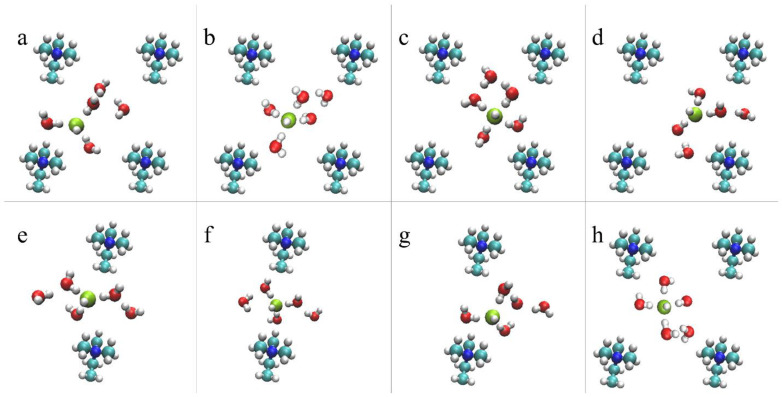
Representative configurations showing the hydroxide ion vehicular diffusion for the AEM model, from a *z*-perspective, in the center of the cell (**a**–**d**) and in the BR (**e**–**h**), including five water molecules from the first and second solvation shells. The colors of the atoms are identical to Figure 1. A green sphere represents the hydroxide ion. See main text for further explanation. Reprinted with permission from Reference [53]. Copyright 2019 American Chemical Society.

For the AEM model under low hydration conditions, vehicular diffusion was found to be the dominant diffusion mechanism [53]. The preference for vehicular over structural diffusion relates to the non-uniform water distribution, which results in an insufficient number of water molecules in the vicinity of the hydroxide ions to promote PT events [53]. Additionally, we find that the hydroxide diffusivity is affected by its location in the cell: the hydroxide ion diffuses relatively freely when located at the center of the cell while its diffusion is restricted when it is located in BRs where the diffusion path between a pair of cations is relatively narrow (see Figure 1). In Figure 4, we propose an idealized vehicular diffusion process and corresponding solvation patterns, while emphasizing specific diffusion requirements for each region. For this purpose, we use O*_2_ of the model AEM along with five water molecules from the first and second solvation shells. Initially, the hydroxide ion is in a stable, “resting”, threefold structure near a cation [52], with two water molecules in the second solvation shell (Figure 4a). Once most of the water molecules are located in the center of the cell, the hydroxide ion drifts towards them via vehicular diffusion, forming a fourfold planar structure in the center of the cell with one water molecule in the second solvation shell (Figure 4b). The hydroxide ion and the five water molecules continue to diffuse via vehicular diffusion towards the nearby cation (Figure 4c), until the hydroxide ion forms, once again, the stable, “resting” threefold structure near the next cation (Figure 4d). To promote hydroxide ion diffusion into the BR, a water molecule must be located near the BR entrance. Once the hydroxide ion is located in the BR, the hydroxide ion forms a threefold tetrahedral structure as part of a well-ordered stable ${\mathrm{OH}}^{-}{{(H}_{2}O)}_{4}$ complex [52], in which three water molecules are part of a threefold tetrahedral structure, where a water molecule is located below the hydroxide ion in the BR, and one water molecules is in the second solvation shell (Figure 4e). Next, the hydroxide ion and the five water molecules diffuse vehicularly through the BR (Figure 4f). We find that the existence of a water molecule above/below the hydroxide ion, as part of the tetrahedral structure, is essential in establishing OH^–^ diffusion through a BR. Additionally, in order to draw the hydroxide ion along the BR, a water molecule is required to be located on the other side of the BR. Once most of the first solvation shell water oxygens diffuse into the next open region, the hydroxide ion is drawn towards the other side of the BR and forms a stable, “resting”, threefold structure near the next cation (Figure 4g). The mechanistic cycle is complete once the threefold hydroxide ion structure drifts towards the center of the cell and reforms into a fourfold planar structure (Figure 4h).

As a vehicular diffusion process requires synchronicity between both of the hydroxide ions and water molecules, the similarity in the diffusivities of both species is expected.

### 4.3. Hydronium Ion Diffusion in the PEM Model System

#### 4.3.1. $H_{3}O^{+}$ Solvation Structure and Dynamical Properties

**Figure 5:**
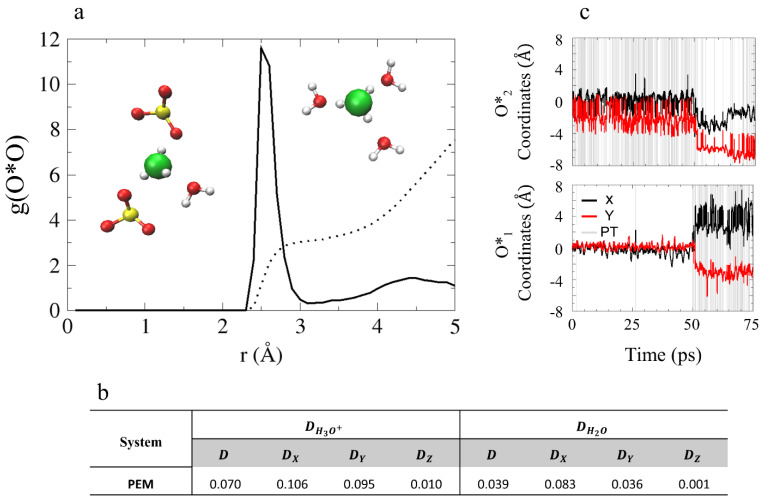
(**a**) O*O RDF for the PEM model, where O* represents the hydronium oxygens and O represents ${\mathrm{SO}}_{3}^{-}$ and water oxygens. The black dotted line represents the obtained CNs. Inset: two examples of hydronium ions in a threefold solvation complex. The colors of the atoms are identical to Figure 1. (**b**) Diffusion constants of the PEM model obtained from the slope of the MSD (presented in SI) in units of Å^2^/ps. (**c**) Hydronium ion oxygen coordinates as a function of time (black and red curves for *x* and *y* coordinates, respectively) for O*_1_ and for O*_2_ during the simulations of model PEM, with PT events are labeled with gray lines (including rattling). Figure (**a**,**c**) are reprinted with permission from Reference [55]. Copyright 2020 Royal Society of Chemistry.

Next, we turn to explore the hydronium ion solvation structure in the model PEM. We start with the O*O RDF and CNs presented in Figure 5 (O* represents the hydronium oxygens and O represents ${\mathrm{SO}}_{3}^{-}$ and water oxygens). As shown, the first solvation shell is located at 2.6 Å, and the CN values are 3.1 and 7.5 for the first and second solvation shells, respectively. Population probabilities for the hydronium ion solvation complexes show that the most common structure is 3A + 0D with 90% (see SI for hydrogen bond (HB) criteria). Additionally, we find that the oxygens taking part in the first solvation shell of the hydronium ion are both ${\mathrm{SO}}_{3}^{-}$ and water oxygens (see inset of Figure 5). Excluding oxygens from the first solvation shell and the ${\mathrm{SO}}_{3}^{-}$ oxygens, the number of water oxygens in the second solvation shell of the hydronium ions is 1.5. This suggests the hydronium ions in the PEM model system are missing a complete second solvation shell as a result of the non-uniform water distribution present in the system. 

In order to enhance the analysis of the hydronium ion diffusion mechanism in this confined environment, we calculate water and hydronium diffusion coefficients for each of the axes (see Figure 5b). Moreover, in Figure 5c we present the coordinates of the hydronium oxygens along the trajectory. We find that the hydronium ion diffusion occurs along the *x*- and *y*-axes, with diffusion coefficients of 0.106 Å^2^/ps and 0.095 Å^2^/ps, respectively. According to Figure 5c, both ions become diffusive (at ~50 ps) after an initial “resting” period. Unlike in the model AEM, the water molecules in the model PEM were found to be non-diffusive, suggesting that water mobility is not necessarily required for $H_{3}O^{+}$ diffusion.

**Figure 6:**
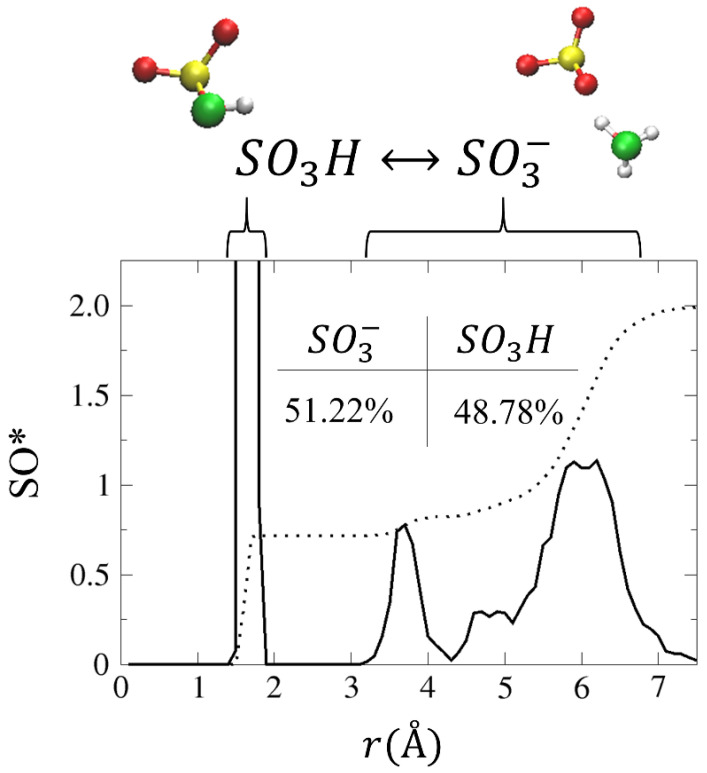
SO* RDF for model PEM. The black dotted lines represent the obtained CNs. Inset: the time in percentage the hydronium ions spent as ${\mathrm{SO}}_{3}^{-}$ and SO_3_H (a detailed description of the calculation can be found in Reference [55]).

In order to gain a better understanding of the conditions that enable the diffusion of the two hydronium ions, we plot in Figure 6 the SO* RDF and CNs. As shown, the first peak, located at ~1.7 Å, corresponds to SO_3_H, in which an H_3_O^+^ has transferred a proton to an ${\mathrm{SO}}_{3}^{-}$, while the second peak is located at ~3.7 Å. The SO* CN values for the first and second solvation shells are 0.7 and 0.8, respectively. This suggests that, as a result of the following reaction: ${\mathrm{SO}}_{3}^{-}{+H}_{3}O^{+}↔{\mathrm{SO}}_{3}{H+H}_{2}O$, a neutral species exists in the system. To verify this, we calculate the percentage of time that the hydronium ions spent as ${\mathrm{SO}}_{3}^{-}$ and SO_3_H, which shows that the hydronium ion appears as SO_3_H for 48.78% of the simulation time (see inset of Figure 6). 

**Figure 7:**
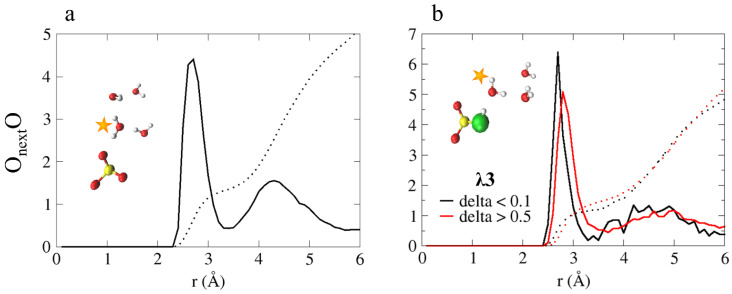
(**a**) O_next_O RDF, in which O_next_ represents the first neighbor oxygen to the ${\mathrm{SO}}_{3}^{-}$ oxygens and O represents all water and hydronium oxygens. (**b**) O_next_O RDFs for δ<0.1 and δ>0.5 (black and red curves, respectively). O_next_ represents the first neighbor oxygen to the ${\mathrm{SO}}_{3}H$ oxygen, and O represents all water and hydronium oxygens. The colored dotted lines represent the obtained coordination numbers. Inset: representative configurations from the AIMD trajectory. The colors of the atoms are identical to Figure 1. Taken with permission from Reference [55]. Copyright 2020 Royal Society of Chemistry.

This leads us to conclude that the protonation state of the sulfonate group is a significant factor in the hydronium ion diffusion mechanism, as the reaction: ${\mathrm{SO}}_{3}^{-}{+H}_{3}O^{+}↔{\mathrm{SO}}_{3}{H+H}_{2}O$, is a key component in the diffusion process. In order to dive a bit deeper into the conditions that enable this reaction, we plot in Figure 7a the O_next_O RDF and CNs, in which O_next_, is the closest water or hydronium oxygen to the ${\mathrm{SO}}_{3}^{-}$ oxygens, and O represents all water and hydronium oxygens. As shown, the first peak is located at 2.7 Å, and the CN values for the first and second solvation shells are 1.3 and 5.1, respectively. The CN values found suggest that before a PT occurs between the anion and a nascent water molecule, the water must have a first solvation shell of one water oxygen and an incomplete second solvation shell. 

In support of this claim, we define a displacement coordinate, $\delta =\left|R_{O_{a}H}-R_{O_{w}H}\right|,$ where $R_{O_{a}H}$ and $R_{O_{b}H}$ are the distances between a shared proton of ${\mathrm{SO}}_{3}H$ and the nearest water oxygen (i.e., O_next_). Values of δ>0.5 are considered to be inactive complexes with respect to PT, while values of δ<0.1 are considered to be “active” and are associated with PT events [41,42,52,69,70]. In Figure 7b, we present the O_next_O RDF and CNs for δ<0.1 and δ>0.5, where, O_next_ represents the first neighbor oxygen to the ${\mathrm{SO}}_{3}H$ oxygen, and O represents water and hydronium oxygens. We find that for δ>0.5, the peak is located at 2.8 Å with a CN value of 1.54, while for δ<0.1, the peak is located at 2.7 Å with a CN value of 1.14. This suggests that in order for the reaction to occur, O_next_ is required to have a CN value of ~1. 

Combining the results presented in Section 4.3.1, we conclude that the factors required to increase the hydronium reactivity in the PEM model relate to the non-uniformity of the water distribution along the membrane, which gives rise to a high probability of obtaining a CN value of ~1 for O_next_, an incomplete second solvation shell for the hydronium ions, and fewer water molecules in the vicinity of the anions oxygens (i.e.,${\;\mathrm{SO}}_{3}^{-}$). 

#### 4.3.2. $H_{3}O^{+}$ Diffusion Mechanism 

**Figure 8:**
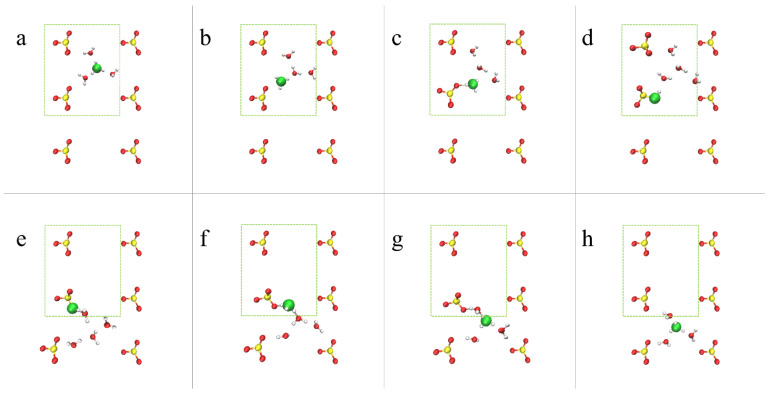
Representative configurations showing the hydronium ion diffusion mechanism for model PEM from a *z*-perspective. The colors of the atoms are identical to Figure 1. A green sphere represents the hydronium ion or ${\mathrm{SO}}_{3}H$. The green rectangles show the primitive simulation cell of the system. See main text for further explanation. Taken with permission from Reference [55]. Copyright 2020 Royal Society of Chemistry.

Based on the results presented in Section 4.3.1 and combined with inspection of configurations from the AIMD trajectory, we propose, in Figure 8, an idealized diffusion mechanism for hydronium ions in the PEM model under idealized hydration conditions (λ=3) [55]. First, the hydronium ion is located in the center of the cell, solvated by three water molecules (Figure 8a). Next, a PT occurs from the hydronium ion to a neighboring water molecule (Figure 8b). A HB is formed between the nascent hydronium ion oxygen and the anion, ${\mathrm{SO}}_{3}^{-}$, while the hydronium has only one water oxygen in its first solvation shell (Figure 8c). Finally, a PT occurs between the hydronium ion and the anion (i.e, ${\mathrm{SO}}_{3}^{-}{+H}_{3}O^{+}$), resulting in ${\mathrm{SO}}_{3}{H+H}_{2}O$ (Figure 8d). This procedure cycles back to the initial condition and restarts, meaning that the next PT will occur once ${\mathrm{SO}}_{3}H$ donates its hydrogen to a neighboring water molecule with a first solvation shell consisting of only one water oxygen (Figure 8f), which will result in ${\mathrm{SO}}_{3}^{-}{+H}_{3}O^{+}$ (see details in Figure 8e–h).

## 5. Discussions and Conclusions

In the present work, we used AIMD simulations to gain an in-depth atomistic perspective of two idealized AEM and PEM models in confined geometries under low hydration conditions (λ = 3, and 4). We found that for both systems, the water distribution within the simulation cell is not uniform. However, the effect of this unique water distribution on the hydroxide and hydronium diffusion mechanisms is fundamentally different. For the AEM model, as long as the hydroxide ions have both first and second solvation shells, the diffusion mechanism is mostly vehicular [53]. However, for the PEM model, hydronium ion diffusion is structural rather than vehicular, with the participations of the anions according to the reaction:$\;{\mathrm{SO}}_{3}^{-}{+H}_{3}O^{+}↔{\mathrm{SO}}_{3}{H+H}_{2}O$ [55].

Comparing the water diffusion, we find that for the AEM model, the diffusion coefficients of the hydroxide ions and the water molecules are similar, as vehicular diffusion requires synchronized diffusion of both species. However, for the PEM model, the water molecules were found to diffuse much slower compared to the hydronium ions, suggesting that water mobility is not necessarily required in the hydronium ion structural diffusion process.

Most importantly, we find that the differences between the AEM and PEM models lie in the essence of the membrane materials. The region between each pair of cations in the AEM system was found to create a BR for hydroxide diffusion, as only specific solvation structures are diffusive, leading to a suppression of hydroxide ion mobility [53]. However, we find that this obstacle is not present in the PEM model, as the anions in the membrane play an active role in the hydronium diffusion mechanism via the reaction ${\mathrm{SO}}_{3}^{-}{+H}_{3}O^{+}↔{\mathrm{SO}}_{3}{H+H}_{2}O$, suggesting that under the right hydration conditions, the presence of the anions in the PEM should promote, rather than suppress, hydronium diffusion [55].

In summary, we believe this work is the first to compare idealized model AEM and PEM in confined environments under low hydration conditions. Using AIMD simulations, we have been able to provide atomistic insight, gaining a fundamental understanding of the uniqueness hydroxide and hydronium ion solvation patterns and diffusion mechanisms in this hitherto unstudied regime. We believe that elucidating the key design principles underlying the atomistic differences between the hydroxide and hydronium ion diffusion mechanisms in model AEMs and PEMs can be a first step toward the discovery and characterization of new, stable, highly conductive membrane materials for use in emerging fuel cell technologies. 

## Supplementary Materials

The following are available online at https://www.mdpi.com/article/10.3390/membranes11050355/s1, Computational methods, Table S1: System parameters, Table S2: Hydroxide and hydronium ions solvation complexes, Figure S1: Radial distribution functions for model PEM. Figure S2: Mean Square Displacements, Figure S3: Example for non-uniform water distribution in model AEM, Figure S4: Example for non-uniform water distribution in model PEM.
