# Supplementary material for: OH− and H3O+ Diffusion in Model AEMs and PEMs at Low Hydration: Insights from Ab Initio Molecular Dynamics
Source: Membranes (Basel). 2021 May 12;11(5):355. doi: 10.3390/membranes11050355 (PMC8151131; doi:10.3390/membranes11050355)
Supplement: Supplementary file 1 [file membranes-11-00355-s001.zip › membranes-1185093-supplementary.pdf]

membranes

Supplementary Information: OH^- and H_3O^+ Diffusion in Model AEMs and PEMs at Low Hydration: Insights from *Ab Initio* Molecular Dynamics

Tamar Zelovich and Mark E. Tuckerman

Computational Methods

We start by constructing the initial structures. For both model anion-exchange membrane (AEM) and proton-exchange membrane (PEM), we first choose the shape of the graphane bilayer (GB) that allows for periodic repetition of the confined structure; in this case, the cell lengths in the periodic directions x and y. We then choose the composition of the linker as $(CH_2)_2$ and attach two tetramethylammonium (TMA) cations to one side of the GB for model AEM and two SO_3^- anions to one side of the GB for model PEM. Next, to achieve the minimum effective volume, we set the distance between the two graphane layers to be 7.3 Å, as this is the maximum possible height of the cations and anions. Finally, we add the selected number of TIP3P water molecules, previously equilibrated in a bulk water simulation, and hydroxide or hydronium ions between the graphane sheets by overlaying the bulk water simulation with the confined structure and selecting molecules with no spatial overlap with the cations or anions.

After constructing the initial structures, *ab initio* Molecular Dynamics (AIMD) simulations [1] were run using the CPMD code [2,3]. We employed the Dispersion-Corrected Atomic Core Pseudopotentials (DCACP) scheme within the Kohn–Sham formulation of Density Functional Theory (DFT) in order to ensure adequate treatment of the dispersion forces, and the B-LYP exchange-correlation functional [4,5], which has proven to be accurate in the treatment of the aqueous hydroxide ion [6]. A plane-wave (PW) basis set was employed to expand the Kohn–Sham orbitals with a cutoff of 80 Ry. The simulations were carried out using the mass of D instead of H for all hydrogen atoms in order to allow a larger time step to be employed and to reduce the importance of nuclear quantum effects. The fictitious mass of the expansion coefficients was taken to be μ=600 a.u., and a time step of 4 a.u. (0.096 fs) was employed for all simulations. Each system was equilibrated at the desired temperature using a massive Nosé–Hoover chain thermostat [7], followed by 15–20 ps of canonical (NVT) dynamics, also using a Nosé–Hoover chain thermostat, finally followed by ~80 ps of microcanonical (NVE) dynamics. For each atomic configuration generated in a simulation, the hydroxide ions in model AEM were identified by finding the two oxygen atoms with a single covalent hydrogen bond, and the hydronium ions in model PEM were identified by finding the two oxygen atoms with three covalent hydrogen bonds. Since each hydrogen can be uniquely assigned to a single oxygen based on the minimum O-H bond length, this assignment is unambiguous. All radial distribution functions (RDFs) were calculated using both the NVT and NVE trajectories, while all dynamic properties were obtained using only the NVE trajectories.

System Parameters

Table S1. System parameters for the two graphane bilayer structures presented in this study.

System	λ	Effective Water Density (g/cm^3)	Cation Spacing (Å)		Cell Geometry (Å)		
			Δx	Δy	x-axis	y-axis	z-axis
**AEM**	4	0.267	10	8.7	10	17.4	7.3
**PEM**	3	0.261	10	6.6	10	13	7.3

For each system, the effective water density is estimated by Equation (1)

$$\varrho = \frac{m_{H_20}[g]}{\left(V_{cell}\text{-}V_{cation/anion}\right)[\mathring{A}^3]} \quad (1)$$

in which the effective volume in the denominator is obtained by subtracting the volume of the cations or anions, $V_{cation/anion}$ (estimated by the size of the cation in its initial configuration) from the volume between the two graphane sheets, V_{cell}. As a result of varying the system parameters (Δx, Δy, and λ), the two systems were constructed with different effective water densities. In order to obtain the highest water density possible, the distance between the two GBs (i.e., Δz) was fixed at 7.3 Å for all systems, the lower bound distance for the cations/anions studied here [8,9].

Hydroxide and Hydronium Ions Solvation Complexes

The population probabilities of different O* solvation complexes for model AEM and PEM are presented in Table S**Error! Reference source not found.**. The Hydrogen Bond (HB) criteria obtained from the OO and O*H RDFs results are as follows: the distance between the oxygen O_a of HB acceptor and the hydrogen H_d and HB donor $R_{O_aH_d} < 2.5$ Å, the distance between the oxygen O_a and oxygen O_d of HB donor $R_{O_aO_d} < 3.5$ Å .

Table S2. Population probabilities of different O* solvation complexes for the two systems.

System	2A + 0D	3A + 0D	3A + 1D	4A + 0D
**AEM**	17.7	67.4	1.5	12.1
**PEM**	-	90.4	-	8.4

Radial Distribution Functions for Model PEM

The OO RDF is presented in Figure **Error! Reference source not found.**a, in which O represents all oxygens in the system but the SO_3^- oxygens. The first peak is located at 2.7 Å. In Figure S1b, we present the OO RDF, in which O represents all oxygens in the system. We find there are two peaks in the first solvation shell. The first peak, at 2.5 Å, corresponds to the OO of SO_3^- oxygens, and the second peak, at 2.8 Å, corresponds to the water oxygens.

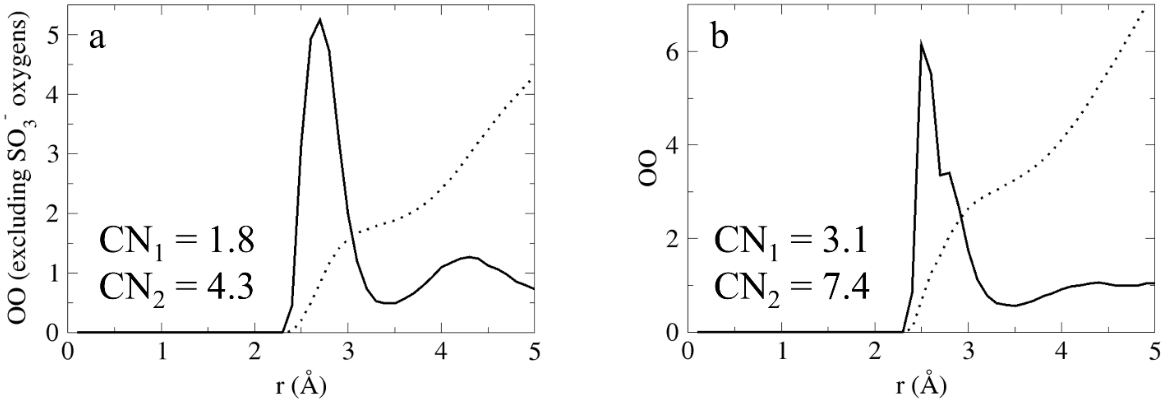

Figure S1. OO Radial distribution functions for model proton-exchange membrane (PEM), in which (**a**) O represents all oxygens in the system, but the SO_3^- oxygens, and (**b**) O represents all oxygens in the system. The dotted lines and the inset of both figures represent the obtained coordination numbers.

Figure S Error! Reference source not found.**: Mean Square Displacements**

Figure S**Error! Reference source not found.** shows the Mean Square Displacements (MSD) as a function of time for the two systems. For each system, the MSD is presented for OH^- and H_3O^+ in the upper panels and H_2O in the lower panels, for the total displacement (black curve) and for each of the axes separately (red, green and blue representing *x*-, *y*- and *z*- axes, respectively). The results presented in Figure S**Error! Reference source not found.** were obtained from the first 10% of the NVE trajectory in order to show the transition to the diffusive-linear behavior appears approximately after 2ps. However, as these confined systems reach the linear regime after 2 ps, the diffusion coefficients are calculated using the slope of the MSD(t) from 2 to 8 ps.

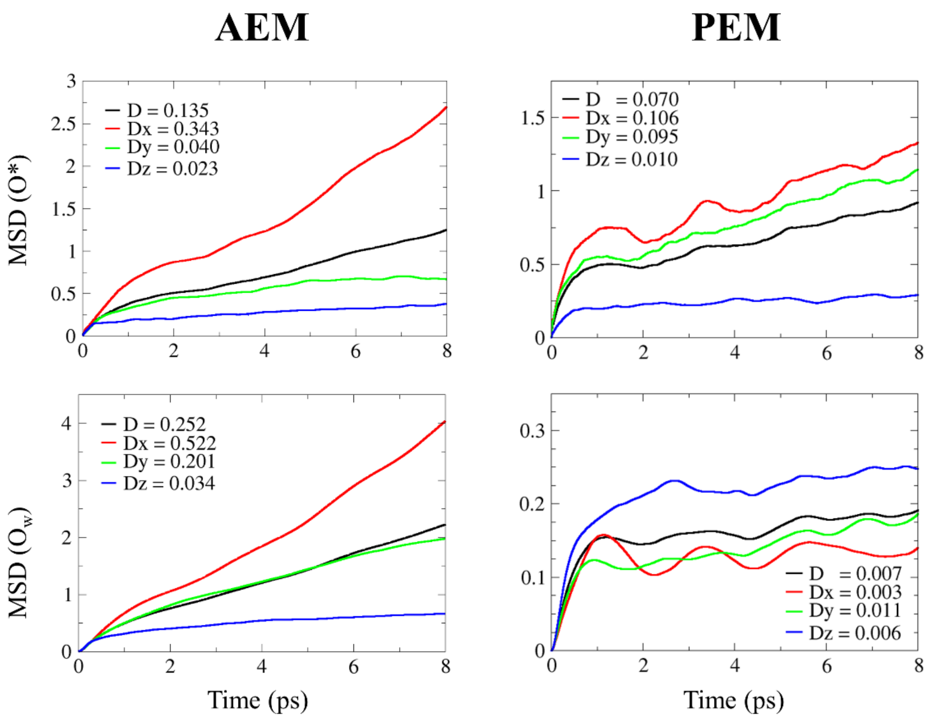

Figure S2. Mean square displacement (MSD) for H_3O^+ and OH^- (upper panels) and H_2O (lower panels) as a function of time, calculated as an average (black curve) and for each of the axes separately (red, green and blue represents *x*-, *y*- and *z*- axes, respectively) for the two systems. Insets:

Diffusion constants obtained from the slope of the MSD in units of Å^2/ps, calculated as an average and for each of the axes separately.

<u>Example for Non-Uniform Water Distribution in Model AEM</u>

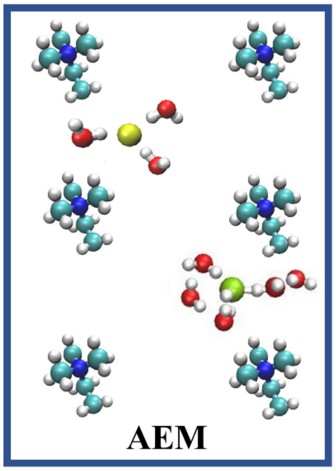

Figure S3. Snapshot of hydroxide ion solvation structures obtained from *ab initio* molecular dynamics (AIMD) trajectories that demonstrate the water distribution, the void regions and the distinct hydroxide ion solvation shells in model anion-exchange membrane (AEM), as presented from a *z*-perspective. Specifically, a non-uniform water distribution is presented, with clusters of three and five water molecules in the vicinity of O^*_1 and O^*_2, respectively, in which only O^*_1 is missing a second solvation shell. Red, white, turquoise, and blue spheres represent O, H, C, and N atoms, respectively. Yellow and green spheres show the position of O^*_1 and O^*_2, respectively. The graphane bilayer atoms (C and H atoms) were removed to better show the water molecules, hydroxide ions, and cation structures. For better view of the systems, we repeat atoms that are beyond the simulation cell boundaries. Taken with permission from Ref. [10]. Copyright 2019 American Chemical Society.

<u>Example for Non-Uniform Water Distribution in Model PEM</u>

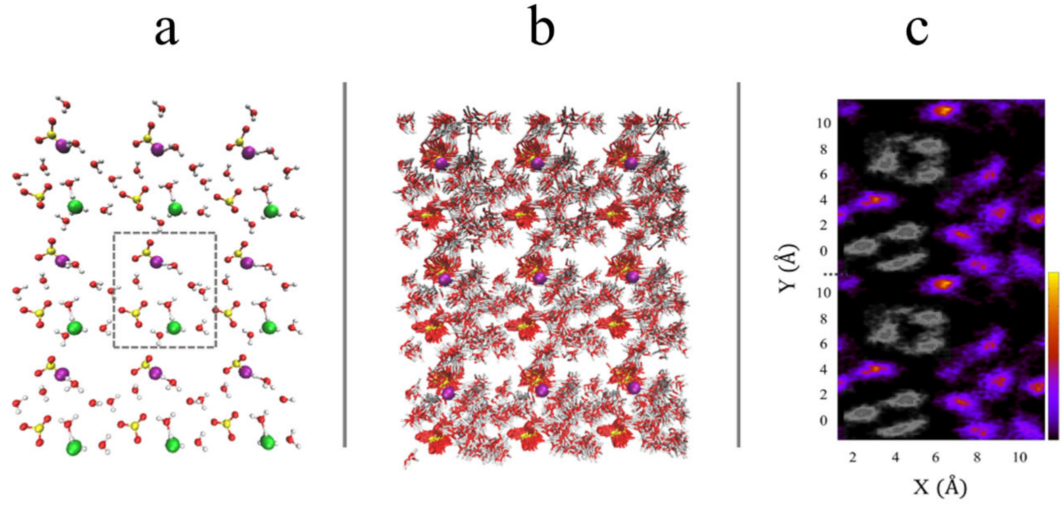

Figure 4. The view of model PEM along the *z*-direction (with the upper and lower graphane sheets removed for clarity). (**a**) Representative snapshots and (**b**) Superposition of configurations sampled every 0.96 ps from the NVE trajectory. The

red, white, turquoise, and yellow spheres represent O, H, C, and S atoms, respectively. The green and purple spheres represent the positions of hydronium ion oxygens. The grey rectangles show the primitive simulation cell of the system. (**c**) Water density profile presented by spatial population of the oxygens in the *xy*-plane. The grey areas represent the locations of the anions throughout the simulations, and the bar color depicts the probability density of the water oxygens locations in the *xy*-plane, normalized according to the number of time steps obtained in the simulation, independent of the *z*-coordinates of the oxygens. The black dotted line shows the primitive simulation cell of the system on the *y*-direction. Taken with permission from Ref. [11]. Copyright 2020 Royal Society of Chemistry.

References

1. Tuckerman, M.E. Ab Initio Molecular Dynamics: Basic Concepts, Current Trends and Novel Applications. *J. Phys. Condens. Matter* **2002**, *14*, R1297–R1355.
2. Marx, D.; Hutter, J. *Ab Initio Molecular Dynamics: Theory and Implementation, in Modern Methods and Algorithms of Quantum Chemistry*; John von Institute for Computing: Juelich, Germany, 2000.
3. Hutter, D.M.J.; Alavi, A.; Deutsch, T.; Bernasconi, M.; Goedecker, S.; Parrinello, M. CPMD, IBM Corporation 1990–2009 and MPI für Festkörperforschung 1997–2001. 2009. Available online: www.cpmd.org (accessed on 20 March 2021).
4. Becke, A.D. Density-functional exchange-energy approximation with correct asymptotic behavior. *Phys. Rev. A* **1988**, *38*, 3098–3100, doi:10.1103/physreva.38.3098.
5. Lee, C.; Yang, W.; Parr, R.G. Development of the Colle-Salvetti correlation-energy formula into a functional of the electron density. *Phys. Rev. B* **1988**, *37*, 785–789, doi:10.1103/physrevb.37.785.
6. Tuckerman, M.E.; Chandra, A.; Marx, D. Structure and Dynamics of OH-(aq). *Acc. Chem. Res.* **2006**, *39*, 151–158, doi:10.1021/ar040207n.
7. Martyna, G.J.; Tuckerman, M.E.; Klein, M.L. Nose-Hoover Chains: The Canonical Ensemble via Continuous Dynamics. *J. Chem. Phys.* **1992**, *97*, 2635–2643.
8. Trigg, E.B.; Gaines, T.W.; Maréchal, M.; Moed, D.E.; Rannou, P.; Wagener, K.B.; Stevens, M.J.; Winey, K.I. Self-assembled highly ordered acid layers in precisely sulfonated polyethylene produce efficient proton transport. *Nat. Mater.* **2018**, *17*, 725–731, doi:10.1038/s41563-018-0097-2.
9. Sepehr, F.; Liu, H.; Luo, X.; Bae, C.; Tuckerman, M.E.; Hickner, M.A.; Paddison, S.J. Mesoscale Simulations of Anion Exchange Membranes Based on Quaternary Ammonium Tethered Triblock Copolymers. *Macromolecules* **2017**, *50*, 4397–4405.
10. Zelovich, T.; Vogt-Maranto, L.; Hickner, M.A.; Paddison, S.J.; Bae, C.; Dekel, D.R.; Tuckerman, M.E. Hydroxide Ion Diffusion in Anion-Exchange Membranes at Low Hydration: Insights from Ab Initio Molecular Dynamics. *Chem. Mater.* **2019**, *31*, 5778–5787, doi:10.1021/acs.chemmater.9b01824.
11. Zelovich, T.; Winey, K.I.; Tuckerman, M.E. Hydronium ion diffusion in model proton exchange membranes at low hydration: Insights from ab initio molecular dynamics. *J. Mater. Chem. A* **2021**, *9*, 2448–2458, doi:10.1039/d0ta10565a.
